# Unveiling the spectrum of sudden cardiac death: a multidisciplinary analysis from the Friuli Venezia Giulia registry

**DOI:** 10.3389/fcvm.2025.1651235

**Published:** 2025-09-26

**Authors:** D. Radaelli, C. Moreschi, R. Bussani, M. Basciu, C. Di Loreto, M. Concato, R. Addobbati, M. Franzin, M. A. Cova, L. Pagnan, G. Girotto, S. Lenarduzzi, B. Spedicati, M. Dal Ferro, G. Sinagra, S. D'Errico

**Affiliations:** ^1^ Department of Medical Surgical and Health Sciences, University of Trieste, Trieste, Italy; ^2^ Azienda Sanitaria Universitaria Giuliano Isontina (ASUGI), University of Trieste, Trieste, Italy; ^3^ Departmental Section of Forensic Medicine, University of Udine, Udine, Italy; ^4^ Department of Medical and Biological Sciences, University of Udine, Udine, Italy; ^5^ Department of Anatomy and Histopathology, ASFO, Pordenone, Italy; ^6^ Institute of Anatomic Pathology, Azienda Sanitaria Universitaria Friuli Centrale, Udine, Italy; ^7^ Department of Medicine (DMED), University of Udine, Udine, Italy; ^8^ Institute for Maternal and Child Health, IRCCS “Burlo Garofolo”, Trieste, Italy

**Keywords:** sudden cardiac death, multidisciplinary approach, autopsy, cardiomyopathy, prevention

## Abstract

**Introduction:**

Sudden cardiac death in individuals aged ≤50 years represents a significant public health issue with diverse aetiologies and age-specific mortality patterns.

**Material and methods:**

This study reports findings from the Friuli Venezia Giulia Sudden Cardiac Death Register in the Young (2021–2024), which employs a multidisciplinary approach integrating autopsy, post-mortem cardiac MRI, toxicology, and genetic testing to investigate sudden and unexpected deaths in the region.

**Results:**

In the first four years a total of 107 cases (mean age 40 ± 10 years; 78% male) were included in the register with ischemic heart disease as the leading cause of death (32%), followed by substance-related fatalities (25%) and sudden arrhythmic death syndrome (SADS, 11%). Substance-related deaths predominated in individuals ≤35 years, while ischemic heart disease was more frequent in those >35 years. Toxicological analyses revealed polysubstance use in most positive cases, with ethanol, benzodiazepines, and methadone most detected. Genetic testing was performed in 14 cases identified pathogenic or likely pathogenic variants in five individuals, mainly involving hypertrophic cardiomyopathy, highlighting the utility of molecular autopsy for familial risk assessment. The registry uncovered a higher burden of substance-related mortality than official statistics, emphasizing underreporting in death certificates. Post-mortem cardiac MRI contributed to detecting myocardial abnormalities, aiding pathological examination.

**Discussion:**

The study underscores the importance of a comprehensive, multidisciplinary registry model for accurate SCD cause determination, improved family screening, and targeted prevention strategies. These findings provide valuable epidemiological and genetic insights relevant to public health interventions, particularly in regions with unique genetic backgrounds such as Friuli Venezia Giulia.

## Introduction

Sudden cardiac death (SCD) represents a major global public health challenge, accounting for approximately 15%–20% of all deaths worldwide (1). The global incidence of SCD varies significantly across different regions and populations. In the United States, the annual incidence is estimated to be between 180,000 and 300,000 cases (2), while in the European Union, recent data from four large population-based registries indicate an average annual incidence ranging from 36.8 to 39.7 per 100,000 population, corresponding to approximately 249,538 SCD cases per year (3). Age significantly impacts SCD incidence, with rates approximately 10 times higher in persons aged 36–49 years compared to those aged 1–35 years (4). Italian data reveal an age-adjusted mortality rate of 0.06 per 100,000 annually (0.10 in males vs. 0.04 in females) among those under 39 years old, with no significant mortality trend changes between 2013 and 2019 (5). Recent trends indicate a concerning increase in SCD-related mortality among adults aged 25–44 years over the past two decades, with notable racial and regional disparities (6). Looking ahead, projections suggest a 90.0% increase in cardiovascular disease prevalence and a 73.4% increase in crude mortality between 2025 and 2050 (7). The observed increase in sudden cardiac death among young adults has been attributed to a complex interplay of factors, including rising rates of substance abuse—particularly opioids and stimulants—persistent racial and regional disparities, and the burden of underlying cardiovascular conditions such as cardiomyopathies and hypertensive heart disease, despite stable overall mortality from traditional ischemic heart disease (6). These multifactorial contributors highlight the urgent need for targeted public health interventions.

In 2020, with the regional Law no. 26, the Friuli-Venezia Giulia region funded the establishment of the regional register of SCD in young people (Friuli-Venezia Giulia Sudden Cardiac Death Register in the Young). The register promotes the systematic investigation of sudden and unexpected deaths in individuals under 50 years of age for whom the cause of death cannot be determined with certainty. The creation of this registry emerged from the recognition of the critical importance of rigorously investigating these tragic events, which are often accepted with resignation without further inquiry. Based on the success of the first three years, on 28 February 2024 with the decree number 7974, the Friuli Venezia Giulia Region financed for other three years the registry. The purpose of this manuscript is to report on the data from the first four years of the Friuli-Venezia Giulia regional register (2021–2024) to characterize the epidemiology of SCD in individuals under 50 years, with specific focus on age-stratified causes (≤35 vs. >35 years), and on the importance of genetic testing, underlining the association between pathogenic variants and fatal outcomes. By combining autopsy findings, post-mortem cardiac MRI, toxicological and molecular analysis, this study aims to unveil the incidence of SCD within the region, identifying age-specific mortality patterns and exploring regionally relevant genetic associations particularly in an Italian region with a high population with Balkan ancestry.

## Materials and methods

The Friuli-Venezia Giulia register aims to systematically collect data on all sudden deaths occurring in apparently healthy individuals aged ≤50 years within the region, with the goal of identifying underlying causes, stratifying epidemiological trends by age and gender, and investigating genetic predispositions to sudden cardiac death. The registry includes sudden and unexpected deaths occurring in apparently healthy individuals aged ≤50 years within the Friuli Venezia Giulia region, defined according to international guidelines as natural deaths of cardiac origin occurring within one hour of symptom onset, or, if unwitnessed, within 24 h of last being seen alive, after the exclusion of non-cardiac causes such as trauma or non-arrhythmogenic intoxications. The age threshold of ≤50 years was selected to focus on a population where non-ischemic causes of SCD, such as inherited cardiomyopathies and channelopathies, are more prevalent, whereas coronary artery disease becomes increasingly dominant with advancing age. This cutoff aligns with prior studies and registries investigating young SCD cohorts, which often apply upper limits of 35–50 years to exclude typical atherosclerotic causes predominant in older adults (8). Mandatory investigations comprise a comprehensive full autopsy, toxicological analysis, post-mortem cardiac magnetic resonance imaging (PM-cMRI) when feasible, detailed histological examination, and genetic testing in selected cases to identify underlying aetiologies and potential hereditary contributions.

It is coordinated by the Cardiology and Legal Medicine Units of the Azienda Sanitaria Universitaria Giuliano Isontina (ASUGI - Trieste) and is based on a multidisciplinary Hub & Spoke organizational model involving various professionals and institutions. It includes a Regional Coordination Centre in Trieste that oversees all registry activities; three Hub Centres for autopsies (Trieste, Udine, and Pordenone); five cardiological Centres (Trieste, Gorizia, Udine, Pordenone, and Tolmezzo); a reference centre for post-mortem cardiac MRI (PM-cMRI) in Trieste; a reference centre for toxicological investigations and a Medical Genetic unit (both in “Burlo Garofolo” Hospital in Trieste). The study protocol and all registry activities were conducted in accordance with ethical standards and received approval from the regional ethics committee (Protocol Number: 0034183/PGEN/ARCS). The Coordination Centre has established standardized “good practices and protocols” based on current guidelines (9, 10) to ensure good coverage across the territory and consistency across the network.

In the first step, general practitioners, emergency physicians, pathologists, prosecutors, or forensic departments notified the Coordination Centre about the death of individuals ≤50 years old. Relevant information, including medical history and circumstances of death, was provided to initiate the process. During autopsy, pathologists were instructed to retain the heart for further analysis, collect samples from all organs and fluid samples for further investigations. These included at least one vial of peripheral blood, central blood, and urine. All specimens were centralized in Trieste, where advanced diagnostic procedures were performed according to the following protocols:

Post-mortem Cardiac MRI (PM-cMRI) was performed ex vivo on hearts removed during autopsy and fixed in formalin. This approach enabled high-resolution imaging of cardiac structures, including myocardial tissue characterization, valve morphology, and wall thickness measurements, providing valuable information complementary to gross and histopathological analyses. Scans were acquired in the Radiology Departement, with a superconductive magnet 1.5T (Philips Gyroscan Achieva, Philips Medical System) or a 3.0 T (Ingenia 3.0T, Philips Medical Systems, Eindhoven, Holland) using a thirty-two channel SENSE cardiac coil. The examination protocol included the following sequences acquired in short axis (SA) and, in pathological cases, long-axis scans were also acquired.

• Turbo Spin Echo (TSE) T1 and T2 Weighted with and without fat suppression using the Dixon technique
• T1 mapping with a Modified Look Locker Inversion recovery (MOLLI);
• T2 mapping with a Black Blood Gradient Spin Echo (BB-m GraSE);
• T2* mapping using a multiecho, gradient-echo pulse sequence.
• 3D b-FFE T2/T1 in SSFP to visualize coronary arteries.

The used parameters are described in Table 1.

**Table 1:** Post-mortem cardiac MRI parameters.

Short axis (acquisition time 20 m)	B-FFE	T1 (Dixon)	T2 (Dixon)	MOLLI (T1 mapping)	M-GRASE (T2 mapping)	T2*MAP (T2 mapping)
TR (msec)	5.5	550	3,420	2.8	1,000	14.4
TE (msec)	1.47	16	100	1.31	14	6.6
FA (°)	90	90	90	35	90	20
Slice thickness (mm)	05	3	3	5	5	5
FOV	200 × 200	160 × 160	160 × 160	170 × 170	170 × 170	170 × 170

Total examination time lasted less than 30 min. Post-processing analysis for T1, T2 and T2* mapping was performed with the IntelliSpace Portal software 9.0 (Philips). Myocardium was automatically segmented into American Heart Association segments (AHA). Motion-correction was performed to exclude partial-volume effect due to formalin near the endocardial wall and epicardial fat. PM-cMRI was used in conjunction with gross and histological analysis to increase diagnostic sensitivity, particularly in challenging myocardial cases. Image interpretation was systematically validated through histopathological correlation: the radiologist provided the pathologist with precise localization data (wall and distance from the valve plane) to enable targeted tissue sampling and examination of specific areas.

Toxicological Analysis was conducted on peripheral blood and urine samples. Initial screening for common drugs of abuse were performed and followed by confirmatory tests using LC-MS/MS tandem mass spectrometry when positive. Blood alcohol content was also measured.

Heart dissection was performed after post-mortem cardiac MRI following the inflow-outflow method (10). Histological examinations were performed using haematoxylin and eosin staining on all organs and a minimum of nine blocks of the heart (apex, three from the LV, two from the septum and three from the right ventricle). Trichromic and immunohistochemistry staining were performed in cases where the initial histological examination indicated the need for a more detailed analysis, such as the evaluation of fibrous tissue extension.

Once all the analyses were performed, all data were returned to the pathologist who submitted a provisional cause of death (COD) according to the criteria showed in Table 2. In cases of diagnostic uncertainty, a second opinion was sought from an expert cardiac pathologist to ensure accuracy in determining COD. If inherited disease was suspected (e.g., sudden arrhythmic death syndrome, specific cardiomyopathy or aortopathy), genetic testing was requested and informed consent was obtained from family members with assistance from the deceased's general practitioner.

**Table 2:** Diagnostic criteria for specific cardiac COD.

Pathology	Macroscopic and microscopic features
Hypertrophic cardiomyopathy	• Increased wall thickness > 15 mm of left ventricle • Increased heart weight (500 g male and 400 g female) • Myocyte hypertrophy and disarray in at least 20% of a slide
Idiopathic left ventricular hypertrophy	• Increased wall thickness > 15 mm of left ventricle • Increased heart weight (500 g male and 400 g female) • No disarray
Myocarditis	• Normal or dilated heart • Diffuse inflammation with myocyte necrosis
Ischaemic heart disease	• Atherosclerosis with estimated luminal narrowing >75% • Sign of acute or chronic infarction
SADS	• Heart macroscopically normal • Normal or contraction band necrosis at histological level
Hypertensive heart disease	• Medical history of hypertension; • Heavy heart with concentric thickening (eventually dilatation); • Exclusion of other causes of cardiomegaly; • Histological evidence of arteriolosclerosis in intramyocardial vessel and other organs; • No other causes of death identified.

Genetic testing was offered to cases meeting at least one of the following criteria indicatives of a potential heritable cardiac condition: (1) morphologically normal heart at autopsy with negative toxicology results, suggesting a likely arrhythmic cause; (2) suspicion of hereditary cardiomyopathy based on gross and histological examination; or (3) suspicion of connective tissue disorder based on macroscopic and microscopic findings. Whole exome sequencing (WES) was reserved for cases lacking a definitive diagnosis after targeted panel testing or presenting complex or atypical phenotypes. This strategy aimed to optimize diagnostic yield while considering resource constraints. Genomic DNA was extracted from whole central blood using the QIAsymphony DSP DNA midi kit v1 and QIAsymphony Robotic Device (Qiagen, Venlo, The Netherlands) according to the manufacturer's instructions. DNA samples were stored at −20°C until use, and their integrity was evaluated with 1% agarose gel electrophoresis. DNA concentration was measured using the QIAxpert Spectrophotometer System (Qiagen, Venlo, The Netherlands). Whole exome sequencing (WES) was performed using the Illumina NextSeq550 instrument (Illumina Inc., San Diego, CA; USA) according to the manufacturer's instructions.

Finally, data were analysed using the EnGenome Expert Variant Interpreter (eVai) software (https://evai.engen ome.com) that allowed variant annotation and interpretation. eVai combines artificial intelligence with the American College of Medical Genetics (ACMG) guidelines (11) to classify and prioritize genomic variants.

Data analysis was focused on 65 genes associated with cardiomyopathies, aortopathies, and channelopathies. The pathogenicity of known genetic variants was evaluated using ClinVar (clinv ar/), Cardiodb (https://www.cardi odb.org/) and The Human Gene Mutation Database (http://www.hgmd.cf.ac.uk/ac/index.php); all variants were classified according to ACMG criteria. Finally, all identified variants were confirmed by Sanger sequencing.

When potentially hereditary cardiovascular causes were identified, first-degree relatives underwent comprehensive cardiac screening. If a pathogenic/likely pathogenic variant was found in the deceased, targeted genetic testing was recommended for first-degree relatives. Relatives testing positive were referred to cardiology departments for clinical follow-up and periodic evaluations based on European Society of Cardiology guidelines (12). Family members who test positive but show no symptoms undergo annual check-ups, while those testing negative with normal baseline screening may discontinue regular monitoring. If autopsy reveals a potentially heritable condition but no pathogenic variant is detected upon genetic testing, first-degree relatives with normal initial screening are advised to undergo periodic assessments every three years from age 18–50.

Throughout this process, referring pathologists and specialists continuously updated the coordination centre which feed the register with new information from autopsy reports, imaging results, toxicology findings, and genetic analyses. This multidisciplinary workflow ensured comprehensive investigation into SCD cases while facilitating family screening programs aimed at preventing future fatal events.

All register data were analysed using Microsoft Excel 365 for statistical evaluation and trend analysis. General population data were extracted from the Italian Institute of Statistics (ISTAT) database (13).

## Results

**Figure 1:**
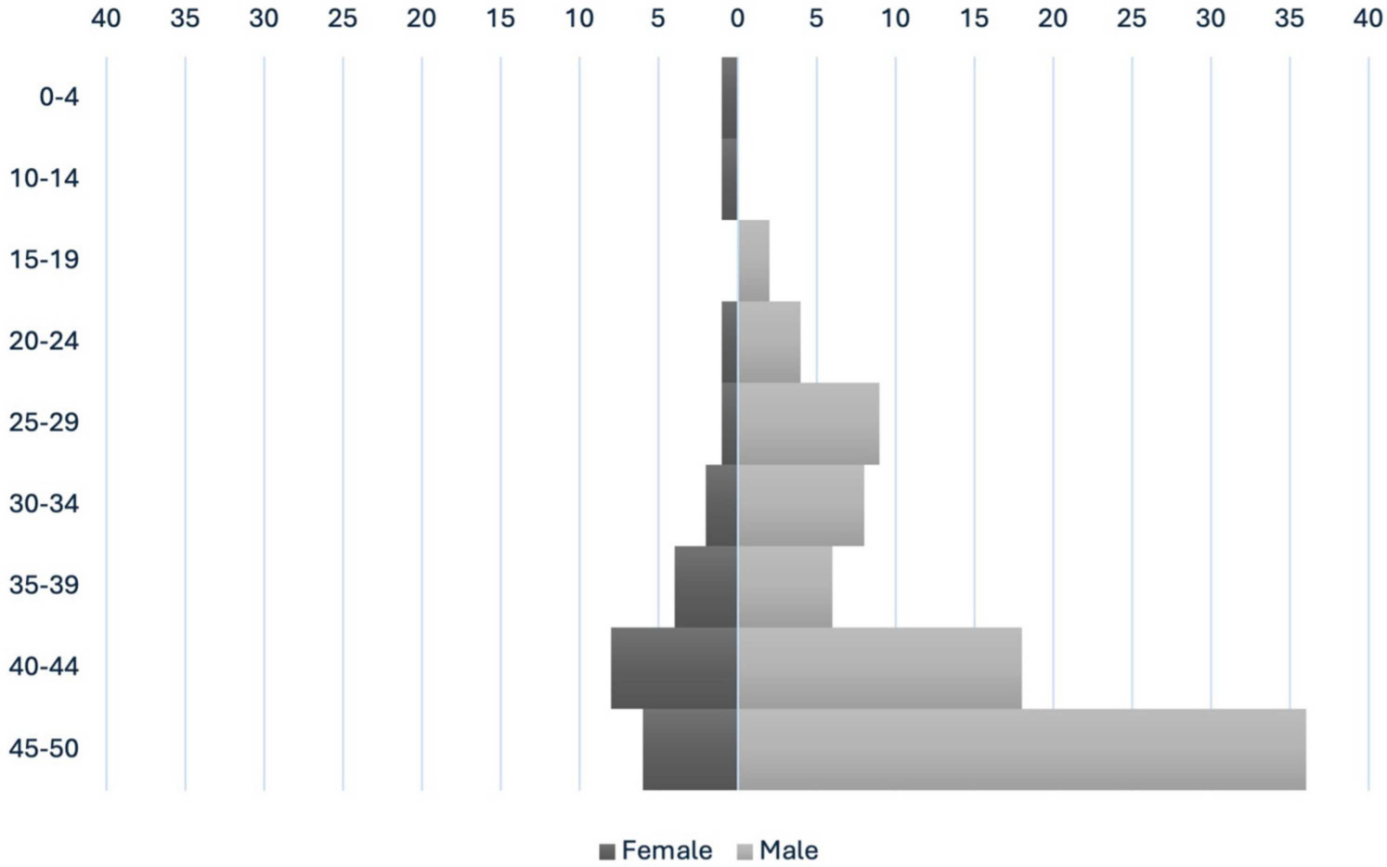
Female vs. male age pyramid.

The Friuli-Venezia Giulia Sudden Cardiac Death Register collected data on 107 cases from January 1st, 2021, to December 31st, 2024. The age of the deceased ranged from 2 to 50 years old, with a mean age of 40 ± 10 years. There was a marked male predominance, with males accounting for 78% of cases (83/107), while females represented 22% (24/107) of the total (Figure 1).

The geographical distribution of cases across the four provinces of Friuli-Venezia Giulia showed that Trieste had the highest number of cases, followed by Udine, Pordenone, and Gorizia. Specifically, Trieste province accounted for 42 cases (39% of the total), with 15 (36% of Trieste cases) being medicolegal autopsies and 27 (64%) being hospital autopsies. Udine province had 30 cases (28%), with 9 (30% of Udine cases) medicolegal autopsies and 21 (70%) hospital autopsies. Pordenone province recorded 24 cases (22%), with 8 (33% of Pordenone cases) medicolegal autopsies and 16 (66%) hospital autopsies. Gorizia province had the fewest cases with 11 (10%), comprising 2 (18% of Gorizia cases) medicolegal autopsies and 10 (82%) hospital autopsies.

Overall, of the 107 autopsies performed, 34 (32%) were medicolegal autopsies requested by judicial authorities, while 73 (68%) were hospital autopsies.

Most deaths (73%, 78/107) occurred at home, while 9% (10/107) happened during or immediately after physical activity. Hospital settings accounted for 6% (6/107) of cases, and the remaining 12% (13/107) occurred in various locations including public places and workplaces. Ten individuals (9%) experienced sudden cardiac death during sport activities, including swimming, soccer, cycling, skiing, and jogging. Curiously, only six individuals (6%) were labelled as sportive by family members. In 17 cases (16%), patients reported cardiac symptoms prior to death, most commonly chest pain and dyspnoea. Family history of sudden cardiac death or related cardiac conditions was documented in 15 cases (14%). Comorbidities were present in a significant proportion of cases, with substance abuse (27%, 29/107), hypertension (18%, 19/107), and obesity (10%, 11/107) being the most frequently reported conditions. Other notable comorbidities included epilepsy, psychiatric disorders, and diabetes mellitus.

A total of 85 PM-cMRI have been performed (79% of the subjects). The mean days between autopsy and PM-cMRI was 32 ± 20 days. In 37 subjects (44%) the radiologist described a significant altered myocardial signal, among which the most common findings were unspecific findings (*n* = 12, 15%) or red flags (myocardial infarction (*n* = 8, 10%), oedema (*n* = 6, 8%), fibrosis (*n* = 5, 6%) and fat infiltration (*n* = 4, 5%).

Toxicological analyses were conducted in 100 cases (91% of the total). Of these, 48 (48%) tested positive on both screening and confirmation analyses, with 29 (29%) considered significant in determining the cause of death. Among the positive cases, 15 individuals (31%) tested positive for a single substance, with a mean age of 42 years and predominantly male distribution (80%, 12 individuals). The remaining 33 cases (69%) tested positive for multiple substances, with a slightly younger mean age of 39 years and a similar male predominance (73%, 24 individuals). The most frequently detected substances were ethanol (*n* = 22, 46%), followed by benzodiazepines (*n* = 17, 36%), methadone (*n* = 15, 31%), cocaine (*n* = 13, 28%), and cannabinoids (*n* = 13, 28%), with morphine and heroin (distinguished by the presence of 6-MAM) found in four cases each (9%). The most common substance combinations were methadone with benzodiazepines (10 subjects, 21%), ethanol with benzodiazepines (8 subjects, 17%), cocaine with cannabinoids (7 subjects, 15%), and methadone with cocaine (5 subjects, 10%).

**Figure 2:**
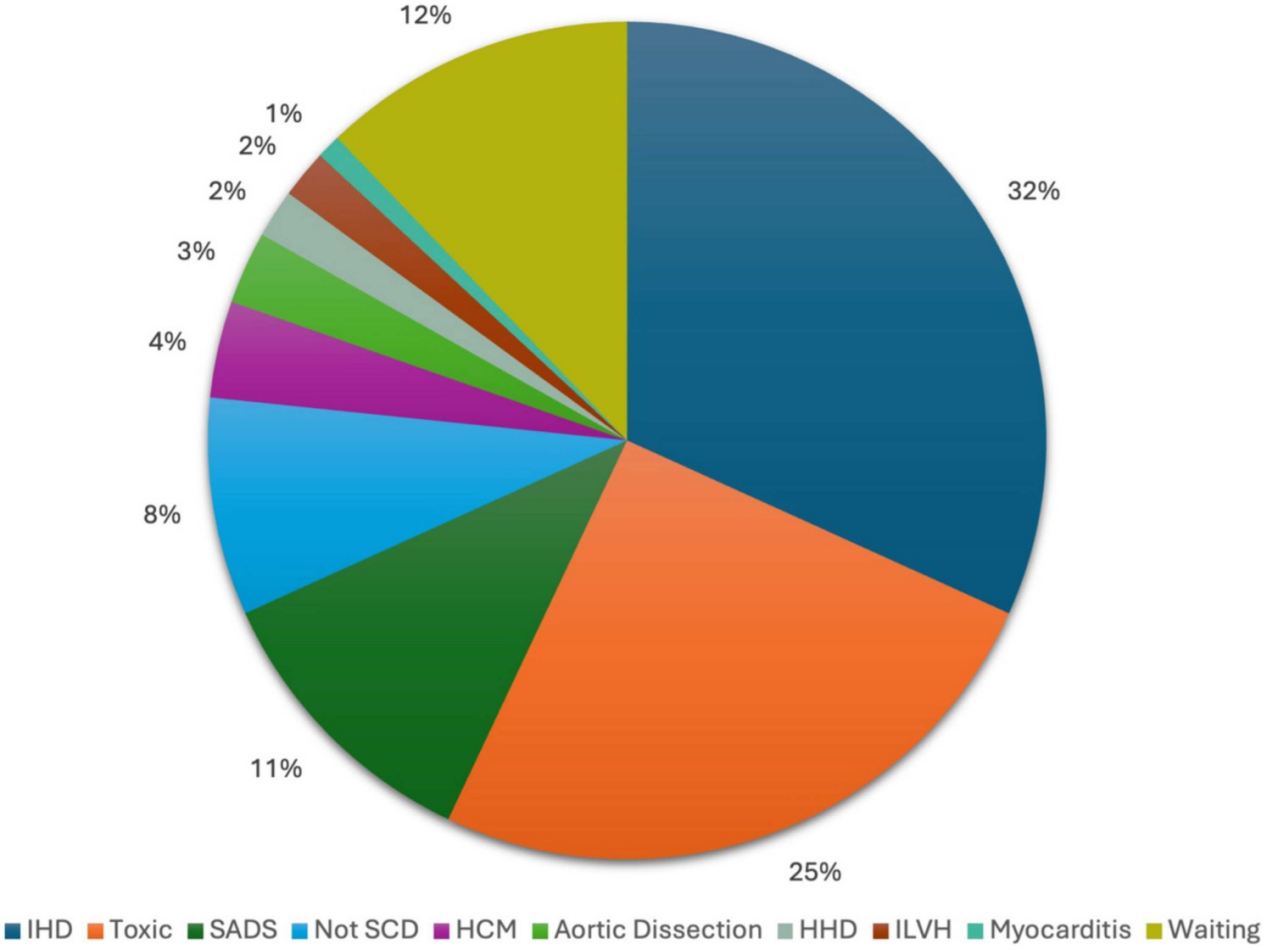
Causes of death determined by march 31st, 2025. IHD, ischaemic heart disease; SADS, Sudden Arrhythmic Death Syndrome; Not SCD, not sudden cardiac death; HCM, hypertrophic cardiomyopathy; HHD, hypertensive heart disease; ILVH, idiopathic left ventricular hypertrophy.

The main causes of death are illustrated in Figure 2. Ischemic heart disease emerged as the most frequent cause, accounting for 32% of cases (*n* = 34). Toxic drug-related deaths were also prevalent, comprising 25% of the cohort (*n* = 27), while a morphologically normal heart - sudden arrhythmic death syndrome (SADS) - was identified in 11% of cases (*n* = 12). Notably, 8% of deaths initially suspected to be cardiac in nature were ultimately attributed to other non-cardiac causes upon further investigation. The remaining causes of SCD were less common and included hypertrophic cardiomyopathy (4%), aortic dissection (3%), hypertensive heart disease (2%), idiopathic left ventricular hypertrophy (2%), and myocarditis (1%). In 13 subjects (12% of the cohort), a definitive cause of death has not yet been established, and additional investigations are still ongoing.

### Age stratification

Upon age stratification, substance-related deaths were most prevalent among individuals aged 35 years and younger (*n* = 30), involving 37% of patients (11 cases), followed by ischemic heart disease and SADS, which accounted for 17% (5 cases) and 14% (4 cases) of deaths, respectively. Hypertrophic cardiomyopathy causes accounted for 10% (3 cases) of deaths, while aortic dissection and idiopathic left ventricular hypertrophy accounted for 3% of demises each (one case both). In contrast, among individuals older than 35 years (*n* = 77), ischemic heart disease emerged as the predominant cause (38% - 29 cases), followed by substance-related deaths and SADS, which explained 21% (16 cases) and 10% (8 cases) of demises, respectively. Non-cardiac causes represented 10% (8 cases), while aortic dissection and hypertensive heart disease accounted for 3% each (2 cases each). Hypertrophic cardiomyopathy, idiopathic left ventricular hypertrophy and myocarditis represented 1% each (one case). Notably, 12% (9 cases) of deaths in this older group remained undetermined, a higher percentage than in the younger cohort.

### Gender stratification

Gender analysis revealed that among males (*n* = 83), ischemic heart disease was the predominant cause at 37% (31 cases), followed by substance-related deaths at 24% (20 cases) and SADS at 9% (7 cases). Non-cardiac causes accounted for 5% (4 cases), while hypertrophic cardiomyopathy represented 4% (3 cases). Less frequent causes included aortic dissection at 3% (2 cases), hypertensive heart disease at 3% (2 cases), and idiopathic left ventricular hypertrophy at 3% (2 case). Myocarditis was identified in 1% (1 case), and 13% (11 cases) remained undetermined. In contrast, among females (*n* = 24), substance-related deaths were more prevalent at 29% (7 cases), followed by SADS at 21% (*n* = 5) and ischemic heart disease (13%; 3 cases). Non-cardiac causes represented 21% (5 cases), while hypertrophic cardiomyopathy accounted for 4% (1 case). Aortic dissection was present in 4% (1 case). No cases of hypertensive heart disease, idiopathic left ventricular hypertrophy or myocarditis were identified among females. Notably, 8% (2 cases) of deaths in females remained undetermined.

### Genetic analyses

Genetic analyses were conducted on 14 subjects who died from aortic dissection, HCM, and SADS. The results are illustrated in Table 3. Variants in SCD-associated genes were identified in eight patients and, noteworthily, two subjects resulted to be carriers of variants in more than one gene of interest. Overall, five Pathogenetic (P) or likely pathogenic (LP) variants and five Variants of Uncertain Significance were identified. Conversely, six subjects tested negative for any genetic variants. WES allowed to identify a definitive molecular diagnosis in all three patients presenting with HCM, identifying P or LP variants in the *MYH7*, *TNNT2*, and *MYBPC3* genes, respectively. Furthermore, in the only patient who died from cardiac tamponade due to aortic dissection, a LP variant was identified in the *FBN1* gene, thus posing a molecular diagnosis of syndromic heart disease, namely Marfan syndrome. Finally, in one case of SADS in a female patient with a history of epilepsy, a LP variant was identified in the *TNNI3* gene. This patient also resulted to be a carrier of a VUS in the *RYR2* gene, which might point towards the presence of a dual molecular diagnosis. Interestingly, an additional female subject with a history of epilepsy was also found to carry a VUS in the *RYR2* gene. Conversely, VUS were mainly identified in subjects presenting with SADS.

**Table 3:** Variants identified through WES analysis in SCD patients.

Patient ID	Gender	Age of death	Clinical phenotype	Gene	Associated disease	Transmission pattern	Dna change	Protein change	Dbsnp	Gnomad_all	Cadd phred	Revel	Acmg/amp classification	References
2	M	31	HCM	*MYH7* (NM_000257.4)	Cardiomyopathy, hypertrophic, 1 (MIM:192600)	AD	c.2609G>A	p.(Arg870His)	rs36211715	0.000009293	26.4	0.853	P	(14)
5	M	44	HCM	*TNNT2* (NM_001001430.2)	Cardiomyopathy, hypertrophic, 2 (MIM:115195)	AD	c.832C>T	p.(Arg278Cys)	rs121964857	0.0005133	24.6	0.631	P	(15)
				*TRPM4* (NM_017636.4)	Progressive familial heart block, type IB (MIM:604559)	AD	c.1195_1202del	p.(Leu399Glyfs*11)	rs780978813	0.000005576	32.0	NA	VUS	NA
26	F	29	SADS	*RYR2* (NM_001035.3)	Ventricular tachycardia, catecholaminergic polymorphic, 1 (MIM:604772)	AD	c.13600C>T	p.(Pro4534Ser)	rs199624074	0.00001431	16.67	0.239	VUS	(16)
				*TNNI3* (NM_000363.5)	Cardiomyopathy, familial restrictive, (MIM:1115210)	AD	c.302A>T	p.(His101Leu)	NA	NA	26.6	0.886	LP	NA
29	M	49	SADS	*CRYAB* (NM_001885.3)	Cardiomyopathy, dilated, 1II (MIM:615184)	AD	c.10G>A	p.(Ala4Thr)	NA	NA	21.4	0.312	VUS	NA
38	F	43	SADS	*RYR2* (NM_001035.2)	Ventricular tachycardia, catecholaminergic polymorphic, 1 (MIM:604772)	AD	c.1082G>A	p.(Cys361Tyr)	rs794728719	NA	25.7	0.861	VUS	(17)
50	F	30	SADS	*TNNI3 K* (NM_015978.3)	Cardiac conduction disease with or without dilated cardiomyopathy (MIM:616117)	AD	c.1340T>C	p.(Leu447Pro)	NA	NA	26.6	0.461	VUS	NA
53	M	32	HCM	*MYBPC3* (NM_000256.3)	Cardiomyopathy, hypertrophic, 4 (MIM:115197)	AD	c.1224-19G>A	NA	rs587776699	0.000008390	14.58	NA	LP	(18)
79	M	20	Aortic Dissection	*FBN1* (NM_000138.5)	Marfan syndrome (MIM:154700)	AD	c.4526A>T	p.(Tyr1509Phe)	NA	NA	25.9	0.694	LP	NA

All variants were detected at the heterozygous state. Clinical phenotype: SADS: Sudden Arrhythmic Death Syndrome. HCM: Hypertrophic Cardiomyopathy. Gene: list of genes carrying the identified variants with NCBI RefSeq accession number of the considered protein-coding transcripts (NM_). Associated disease: genetic disorder nomenclature according to the Online Mendelian Inheritance in Man® (OMIM®) database. Transmission pattern: AD: autosomal dominant. DNA change and Protein change: variant description according to the Human Genome Variation Society (HGVS) nomenclature guidelines. dbsnp: variant accession code according to the Single Nucleotide Polymorphism Database public archive for genetic variation. GnomAD_ALL: allele frequency. CADD_PHRED, REVEL: variant effect evaluated via in silico prediction tools. ACMG/AMP classification: variant pathogenicity according to ACMG/AMP guidelines. VUS: Variant of Uncertain Significance. LP: Likely Pathogenetic. P: Pathogenic. References: PubMed unique IDentifier (PMID) of publications reporting each variant. NA: not available.

So far, seven families have been enrolled for clinical cardiological screening, with four of them remaining under ongoing cardiological care. Notably, in four families, relatives carrying variants of uncertain significance (VUS) loke those identified in the probands were detected and subsequently taken in charge by the cardiology department for personalized follow-up and monitoring. This cascade approach has facilitated targeted surveillance and underscores the clinical relevance of genetic findings beyond the initial diagnosis.

## Discussion

The present study provides a comprehensive analysis of SCD cases in the Friuli-Venezia Giulia region, based on 107 autopsies conducted between January 1st, 2021, and December 31st, 2024. Our results, while offering a promising overview of the region's demographic landscape, highlight the necessity for larger cohorts and prospective analyses to more clearly elucidate the impact of genetics on sudden cardiac death in young people.

Our cohort demonstrated a clear male predominance (78%), consistent with previous studies on SCD (19). The geographical distribution showed Trieste province accounting for the highest proportion of cases (39%), followed by Udine (28%), Pordenone (23%), and Gorizia (10%). The higher proportion of SCD cases in Trieste reflects its role as a central hub for registry activities and advanced diagnostic procedures, while reflecting a potential selection bias where cases may be underreported or missed in other provinces. These findings highlight the need for standardized protocols across all provinces to ensure equitable case detection and reporting.

Ischemic heart disease emerged as the predominant cause of SCD, accounting for 32% of all cases. This high prevalence aligns with the mean age observed in our cohort (40 ± 10 years), highlighting the significant impact of coronary artery disease even in relatively young populations. The results suggest the critical need for early detection and aggressive management of cardiovascular risk factors in young individuals, possibly implementing genetic screening for familiar hypercholesterolemia when critical stenosis is found in a young individual who die suddenly (20).

The high prevalence of toxic drug-related deaths (25%) is particularly concerning and points to the need for enhanced substance abuse prevention and intervention strategies. In this group autopsy findings revealed that the hearts were morphologically normal or exhibited only minimal pathological changes. These included mild valvular thickening, evidence of resolved endocarditis, and non-critical coronary artery stenoses. No significant cardiomyopathies or arrhythmogenic substrates were detected.

Toxicological analyses in this cohort revealed that nearly half (48%) of tested cases were positive for psychoactive substances, with the majority (69%) involving polysubstance use. Ethanol, benzodiazepines, and methadone emerged as the most frequently detected substances, reflecting patterns consistent with broader substance use trends. Notably, methadone-benzodiazepine co-ingestion was identified in 21% of positive cases, while ethanol-benzodiazepine combinations accounted for 17%. These findings underscore a critical public health challenge: the widespread and hazardous practice of combining central nervous system (CNS) depressants, which synergistically exacerbate respiratory depression, cardiac arrhythmias, and fatal overdose risk (21). The high prevalence of methadone-benzodiazepine use aligns with prior evidence demonstrating that concurrent use of opioids and benzodiazepines significantly elevates the risk of QT interval prolongation and torsades de pointes in predisposed patient even at therapeutic levels (22, 23).

Similarly, ethanol-benzodiazepine co-ingestion amplifies GABAergic neurotransmission, leading to profound sedation and respiratory failure, a mechanism well-documented in overdose-related fatalities (24). Studies from Finland have shown that elevated blood alcohol levels are common in SCD victims due to non-ischemic heart disease, especially in males (45% vs. 31% in females), suggesting that recent alcohol consumption might contribute to subsequent SCD in many non-ischemic cases (24).

These findings resonate with studies highlighting polysubstance use as a key driver of mortality in both SCD and overdose populations (25, 26). An Australian prospective cohort study found that 28.8% of confirmed cardiac causes of out-of-hospital cardiac arrest had positive toxicology testing/screening for illicit drugs, with patients showing higher rates of cardiomegaly (76.5% vs. 57.5%), coronary disease, and cardiomyopathy (25). The repetitive detection of these combinations suggests health system issues, such as inadequate access to mental health services, harm reduction programs, or clinician awareness of polypharmacy risks. Furthermore, these data emphasize the need for targeted interventions, including routine toxicological screening in SCD cases, education on safe prescribing practices, and expanded access to opioid agonist therapies with reduced cardiac risk profiles (26).

Curiously, our findings reveal a significant discrepancy between the number of drug-related deaths reported in our study and the official statistics from the ISTAT database for the Friuli-Venezia Giulia region in 2021 and 2022 (13). While ISTAT reported only five and seven deaths in the general population due to drug abuse for these respective years, our registry identified a higher number of toxic drug-related deaths (*n* = 7 for 2021 and *n* = 11 for 2022) within a much more restricted cohort comprising only subjects younger than 50 years old. This stark contrast underscores the potential underreporting of drug-related fatalities in official statistics when based solely on death certificates.

This discrepancy likely stems from several methodological factors. First, our registry employs comprehensive toxicological screening and post-mortem examinations that can detect substance involvement even when not apparent from external examination or medical history (27). Second, death certificates often classify the immediate cause of death (e.g., cardiac arrest, respiratory failure) without identifying the underlying substance contribution, particularly in cases where polysubstance use complicates the clinical picture (27). Third, social stigma surrounding substance use may influence reporting practices, with families or healthcare providers reluctant to document drug involvement without definitive evidence (28, 29). The implications of this underreporting are profound for public health surveillance and intervention planning (30). Accurate quantification of substance-related mortality is essential for appropriate resource allocation, policy development, and evaluation of harm reduction strategies (31). Our findings suggest that current surveillance systems may substantially underestimate the true burden of substance-related mortality, particularly among younger populations. This highlights the need for standardized toxicological screening protocols in unexpected deaths and improved integration between forensic data and public health reporting systems to capture the full spectrum of substance-related mortality. This discrepancy highlights the critical importance of comprehensive post-mortem investigations, including thorough autopsies and toxicological analyses, in accurately determining the cause of death. Without such comprehensive examinations, the true impact of substance abuse on mortality rates may be significantly underestimated, potentially hindering effective public health interventions and policy-making efforts aimed at addressing this critical issue. Additionally, failure to conduct toxicological screening may result in the misclassification of these cases as SADS (19). Such misdiagnosis could trigger unnecessary genetic testing, leading to unwarranted emotional distress for families and futile expenditures for healthcare systems.

Notably, our study's molecular autopsy yield stands at an impressive 35%, placing it at the higher end of the “yield-spectrum” compared to previous reports (32–34). This further emphasizes the effectiveness of genetic testing in identifying potential causes of sudden death and selecting which family groups should undergo cardiological screening and genetic counselling.

The identification of SADS in 11% of cases underscores the vital importance of genetic testing and family screening in preventing SCD. Notably, none of the deceased individuals in this cohort had a prior diagnosis of cardiovascular disease, with SCD representing the first manifestation of their underlying condition. Three subjects had a documented history of epilepsy, and one had a diagnosis of psychosis. Unfortunately, only 14 families provided consent for genetic testing, limiting our ability to comprehensively assess hereditary factors in sudden cardiac death cases. This relatively low participation rate may reflect various barriers, including grief-related reluctance and insufficient awareness of the potential implications of genetic findings. Notably, we also encountered consistent refusal in certain families belonging to minority groups, even after extensive efforts to explain the benefits of genetic testing and provide support. Such refusals suggest that additional, potentially culturally driven factors may further impede enrolment in these communities, emphasizing the need for adapted communication strategies and enhanced outreach to improve participation in genetic evaluation. Despite these challenges, the genetic analyses performed on this subset revealed valuable insights, including the identification of pathogenic and likely pathogenic variants in genes linked to cardiomyopathies and channelopathies in five SCD cases. Specifically, all three HCM cases were molecularly solved, highlighting once again the high diagnostic yield of Next-Generation Sequencing analyses in young patients presenting with this phenotype (35, 36). Causative variants were identified in *MYH7* and *MYBPC3*, which are the most commonly mutated genes in HCM subjects (37), and *TNNT2*, whose pathogenic or likely pathogenic variants are relatively rare, being identified in only 4% of HCM cases (38). A LP variant was identified in the *FBN1* gene, which is associated with Marfan syndrome (MIM: # 154700), in a patient who died of aortic dissection: this result underlines how syndromic features may sometimes blend into the wide spectrum of physiological findings, thus hampering the recognition of rare disorders. Conversely, a precise clinical diagnosis is fundamental to provide patients with the appropriate follow-up and prevent life-threatening complications (39). Identifying a molecular diagnosis in SADS patients has proven particularly challenging: indeed, only one LP variant in the *TNNI3* gene was identified in one patient out of four SADS cases. Two of the remaining patients harboured VUS in the *TNNI3K* and *CRYAB* genes: the identification of VUS in SCD-associated genes gives rise to huge uncertainty regarding the appropriate clinical management of other family members and demands for reinterpretation of genetic tests results in time (40). An intriguing finding is represented by the presence of *RYR2* variants in two female subjects with a history of epilepsy. Variants in the *RYR2* genes are associated with Ventricular tachycardia, catecholaminergic polymorphic, 1 (MIM: # 604772), a condition that, even in the absence of morphological and electrocardiographic alterations, may directly present with SCD The findings offer valuable insights into the demographic characteristics, causes of death, and the role of genetic factors in SCD during physical or emotional stress (41). This causes significant difficulties in the identification of at-risk patients, making the application of meaningful preventive strategies extremely hard. Furthermore, considering the epilepsy history of both patients, further exploration of the potential connection between cardiac channelopathies and neurological disorders should be warranted (42). Indeed, this finding might further support the hypothesis that channelopathy-related arrhythmias play a central role in the aetiopathogenesis of Sudden Unexpected Death in Epilepsy (SUDEP) and suggests how the molecular autopsy should be a fundamental step of post-mortem evaluations in this group of patients (43). An expansion of the cohort of SUDEP patients and further functional studies are mandatory to better explore the molecular basis of this not-so-rare event, whose pathophysiology remains widely unexplored.

Interestingly, six subjects tested negative at the molecular level, highlighting the challenges of clinical evaluation. Inherited cardiovascular disorders are marked by significant clinical heterogeneity, and often only partial medical histories are available. Our understanding remains limited, as many genes involved in cardiac disorders are still unknown despite advances in genomic research. Technological limitations persist, with certain regions of the human genome difficult to analyse and thus poorly covered. The role of structural variants also warrants consideration. Lastly, some pathologies might not follow Mendelian inheritance patterns, raising questions about their genetic basis.

When stratifying our data by age, we observed that substance-related deaths were most prevalent (37%) in individuals aged 35 years and younger, followed by ischemic heart disease (17%) and SADS (14%). In contrast, among individuals older than 35 years, ischemic heart disease was the predominant cause (38%), followed by substance-related deaths (21%). This age-related pattern aligns with established epidemiological trends showing that SCD in young adults is frequently associated with non-ischemic causes, while coronary artery disease becomes increasingly dominant with advancing age (4). The high prevalence of substance-related deaths in our younger cohort mirrors recent data showing rising trends in substance use mortality among younger populations, highlighting the need for age-specific prevention strategies (44).

Gender analysis revealed notable differences in causes of death. Among males, ischemic heart disease was the predominant cause (37%), followed by substance-related deaths (24%). This pattern corresponds with established cardiovascular research demonstrating that men generally develop coronary artery disease earlier than women and have a higher predisposition for SCD, especially when combined with family history (45). In contrast, among females, substance-related deaths were more prevalent (29%), followed by SADS (21%) and ischemic heart disease (13%). These gender-based differences underscore the importance of sex-specific approaches to SCD prevention (46).

The PM-cMRI initially presented challenges. Early in the registry, radiologists often refrained from interpreting identified abnormalities because formalin fixation altered myocardial signals, complicating the semeiotic analysis. However, as experience grew, collaboration with pathologists led to the development of a specific semiology and a standardized reporting protocol for PM-cMRI. This protocol includes measuring distances from the valve plane when altered signals are detected, enabling pathologists to precisely study affected areas both macroscopically and microscopically. Post-mortem cardiac MRI proved to be a valuable tool in guiding the macroscopic examination of the heart, enabling targeted assessment of specific regions and more precise identification of areas warranting detailed histological analysis. This integrated approach enhanced the overall accuracy and efficiency of the post-mortem investigation. Interestingly, in seven subjects who showed altered signal, both in T1 and in T2 weighted images, with vascular distribution and died from ischemia due to severe stenosis or complete occlusion, the myocardium appeared unremarkable upon dissection at both macroscopic and microscopic levels. This could suggests that PM-cMRI may effectively detect ischemic myocardium in its early stages (47). However, these preliminary findings require further validation through additional comprehensive studies to establish the technique's diagnostic accuracy and clinical utility in forensic and pathological settings. Additionally, although in 4 subjects fat infiltration was detected on PM-cMRI, the subjects did not exhibit fibro-fatty replacement at the histological level, preventing a diagnosis of arrhythmogenic cardiomyopathy (ACM). Curiously, the causes of death in our cohort differ from those reported in the neighbouring Veneto region. Specifically, while previous studies in Veneto documented arrhythmogenic and dilated cardiomyopathies as causes of sudden cardiac death in approximately 10% and 4% of cases respectively (48), our cohort exhibited a lower prevalence of these cardiomyopathies. This discrepancy may be attributable to differences in observation periods and inclusion criteria. Furthermore, despite the geographical proximity, the populations of Friuli Venezia Giulia and Veneto differ in ethnic composition. In particular, the provinces of Gorizia and Trieste in Friuli Venezia Giulia have a significant proportion of individuals with Balkan ancestry. This distinct genetic and ethnic background could contribute to variations in the prevalence and types of cardiomyopathies and other causes of sudden cardiac death observed between these regions.

Similarly, international data highlight the heterogeneity in the epidemiology of sudden cardiac death across populations. The Danish nationwide study by Risgaard et al. (4) reported coronary artery disease as the leading cause of sudden cardiac death in persons aged 1–49 years, accounting for approximately 36% of autopsied cases, followed by a high proportion of sudden unexplained deaths (31%). While inherited cardiomyopathies and arrhythmogenic syndromes were also significant contributors, toxicological factors played a lesser role compared to our registry, which emphasizes substance abuse-related deaths alongside genetic and structural cardiac abnormalities. These differences underscore the complex interplay of genetic, environmental, and socio-cultural determinants influencing regional patterns of sudden cardiac death, reinforcing the necessity for tailored prevention and diagnostic approaches.

According to data from ISTAT, the Friuli-Venezia Giulia region had 612,372 residents aged 50 years or younger in 2021. When incorporated into the registry, which recorded 12 cardiac deaths in the same year, the estimated incidence of SCD in the region was 1.96 per 100,000 inhabitants per year. In subsequent years, the incidence climbed to 2.46 per 100,000 in 2022 (total population of 610,279 and 15 reported cardiac deaths) and 3.14 per 100,000 in 2023 (604,363 and 19 reported cardiac deaths). The observed 60% increase in incidence could be attributed to the limited number of autopsies included in the registry, which represents a significant limitation. For instance, ISTAT data indicates that 3,509 individuals died in the Friuli-Venezia Giulia region in 2021. However, only 0.60% of these cases were included in the registry. Similar results were observed in subsequent years, with the inclusion rate approaching 1% in 2023 (0.94%). This low coverage stems primarily from selective case inclusion based on referral pathways, consent acquisition, and resource availability for comprehensive post-mortem investigations such as post-mortem cardiac MRI and genetic testing. Consequently, the sample may not be fully representative of the whole population, potentially introducing selection bias favouring cases with clearer diagnostic pathways or specific referral patterns. Moreover, the underrepresentation of certain subgroups may limit the generalizability of epidemiological findings and the accurate estimation of the true incidence and etiological spectrum of sudden cardiac death in the region. Achieving broader case capture through enhanced collaboration with regional mortuary services, standardized protocols across healthcare and forensic centres, and improved family engagement is essential for overcoming this limitation and increasing the comprehensiveness and validity of future registry data.

Another limitation lies in the challenges of obtaining complete information from pathologists. In some cases, not all necessary details were provided, such as measurements of the left ventricle wall. Due to incomplete availability of anthropometric data, weight and height measurements necessary for the calculation of body mass index (BMI) were not systematically collected for all subjects. Consequently, BMI analysis was not performed in this study. This limitation is acknowledged and highlights the need for standardized collection of such data in future registry updates.

Additionally, not all cases underwent toxicological analyses, which are legally mandated. To address these issues, the coordination centre plans to implement new strategies and protocols to enhance data collection and ensure more comprehensive reporting. Greater involvement of general practitioners and pathologists will be crucial in this process.

A new software system is being developed to facilitate data collection by allowing all physicians to access and complete their respective sections while indicating which data to collect. Issues with obtaining consent for genetic testing were also identified, as not all eligible subjects had family consent for analysis. Potential solutions include outreach programs and educational initiatives to help families understand the importance of genetic testing, potentially increasing participation.

Lastly, the average time between autopsy and PM-cMRI was 32 ± 20 days, which is too lengthy for both pathologists and families. This delay results from limited availability of machine slots. Prolonged formalin fixation during this interval may adversely affect tissue characteristics, leading to potential degradation of MRI signal quality and the introduction of imaging artifacts, which can complicate interpretation. While histopathological correlation was used to mitigate misinterpretations, this delay remains a methodological limitation. Possible solutions include increasing the availability of instruments or sharing the MRI protocol with other regional trusts while supervising the reporting process.

## Conclusions

In conclusion, this study highlights the critical insights gained from a comprehensive analysis of SCD cases in the Friuli Venezia Giulia region. The findings underscore the importance of a multidisciplinary approach, as adopted by the registry, in understanding the complexities of SCD. By integrating results from various investigations, including genetic testing, PM-cMRI, and autopsies, we can achieve a complete understanding of the causes of death and identify potential risks for family members. This systematic and multidisciplinary approach to address sudden death in young individuals serves as a unique model at the national level. It is supported by dedicated regional resources aimed at implementing the registry and enhancing existing professional expertise within the territory.

Such a model not only improves our understanding of SCD but also facilitates early detection and management of cardiovascular risk factors, ultimately contributing to better public health outcomes. Recent evidence has demonstrated that ethnicity has significant associations with SCD risk factors, presenting rhythms, and outcomes (49–51). Ethnic differences in SCD may be influenced by cultural, social, genetic, and environmental factors, including health behaviours, comorbidity burden, and access to healthcare. The creation of an international registry that includes neighbouring Balkan countries would provide valuable comparative data on ethnic differences in SCD patterns, lifestyle factors, and genetic predisposition. This is particularly relevant for the provinces of Gorizia and Trieste, which have historically been influenced by Balkan populations. Moving forward, we envision expanding our collaborative network to establish standardized protocols across diverse populations, ultimately contributing to more targeted prevention strategies and personalized approaches to reduce the burden of sudden cardiac death worldwide.

## Data Availability

The datasets presented in this article are not readily available due to the sensitive nature of the topic addressed in our article, and in compliance with applicable privacy regulations and legal constraints. Requests to access the datasets should be directed to the corresponding author.
